# Inheritance of Acquired Traits in Insects and Other Animals and the Epigenetic Mechanisms That Break the Weismann Barrier

**DOI:** 10.3390/jdb9040041

**Published:** 2021-10-07

**Authors:** V. Gowri, Antónia Monteiro

**Affiliations:** 1 Department of Biological Sciences, National University of Singapore, Singapore 117543, Singapore; antonia.monteiro@nus.edu.sg; 2 Science Division, Yale-NUS College, Singapore 138609, Singapore

**Keywords:** Weismann barrier, inheritance of acquired traits, transgenerational epigenetic inheritance, epigenetics, genetic assimilation, insects

## Abstract

The credibility of the Weismann barrier has come into question. Several studies in various animal systems, from mice to worms, have shown that novel environmental stimuli can generate an altered developmental or behavioral trait that can be transmitted to offspring of the following generation. Recently, insects have become ideal models to study the inheritance of acquired traits. This is because insects can be reared in high numbers at low cost, they have short generation times and produce abundant offspring. Numerous studies have shown that an insect can modify its phenotype in response to a novel stimulus to aid its survival, and also that this modified phenotypic trait can be inherited by its offspring. Epigenetic mechanisms are likely at play but, most studies do not address the mechanisms that underlie the inheritance of acquired traits in insects. Here we first review general epigenetic mechanisms such as DNA methylation, histone acetylation and small noncoding RNAs that have been implicated in the transmission of acquired traits in animals, then we focus on the few insect studies in which these mechanisms have been investigated.

## 1. Introduction

Ideas and evidence around the inheritance of acquired traits have a tortuous history. In 1809, Jean-Baptiste Lamarck put forth a theory of inheritance, and Charles Darwin, in 1868, presented his Pangenesis theory [1]. Both theories claimed that traits or characteristics acquired during an organism’s life can be inherited by the next generation. However, in 1885 August Weismann disregarded these initial ideas on the inheritance of acquired traits by proposing that only the germ cells, but not the somatic cells, are capable of carrying hereditary information. Furthermore, he proposed that influences on somatic cells cannot be passed to the germ cells during an organism’s lifetime or be inherited by the offspring. This concept of a “Weissman’s barrier” was supported by Hugo de Vries’s theory of Intracellular Pangenesis in 1889, which proposed that particles of inheritance cannot be transferred from the somatic cells to the germ cells [2]. Thus, the idea of inheritance of learned or acquired traits lost steam. By the 1990s, however, several scientists published data suggesting that the environment can influence the phenotype of an individual, and that this initially environmentally-induced phenotype can be transmitted to the offspring.

The inheritance of acquired traits has now been widely observed across animals. One of the common textbook examples is that of *Daphnia cucullata*. These water fleas develop taller helmets in the presence of *Chaoborus glassworm* kairomones, the chemical signal of their predators [3]. The taller helmets make predation of the *Daphnia* more difficult. This adaptive defense phenotype was also found in the offspring of the exposed generation, who were not exposed to the kairomones themselves [4]. Another example comes from an experiment where female mice were fed a diet that was rich in vitamin B12, betaine, choline and folic acid, before, during, and after pregnancy. These females gave birth to lean mice with brown fur, containing the pigment eumelanin, that had a normal lifespan. Mothers that were not fed this special diet, however, had obese offspring with yellow fur containing a different pigment, pheomelanin. These mothers also had a higher risk of diabetes and cancer. This shows that diet, which is an external factor, influences a variety of patterns of gene expression, without altering gene sequences. The methyl groups from the rich diet attached to the Agouti gene, which led to its silence, and this silenced gene condition was inherited by the offspring [5]. Pregnant guinea pigs injected with six doses of betamethasone, a glucocorticoid drug, throughout their prenatal period, suffered changes in the functions of their pituitary and adrenal glands that were sex-specific [6]. The young male offspring were more affected and had higher physiological and behavioral abnormalities compared to the females. These abnormalities in the offspring were passed on to the next generation [7]. In another popular example, mice that were exposed to a neutral fruity odor coupled with a mild electric shock exhibited a startle behavior when exposed to that same odor later in life. This behavior was observed in the two subsequent offspring generations. The untrained offspring startled upon the odor exposure without experiencing the shock [8]. In a final example, *Caenorhabditis elegans* worms acquired resistance to oxidative stress and proteotoxicity (toxicity caused by proteins that are misfolded or damaged) when exposed to heavy metals or starved for a day. This adaptation confers a survival advantage, and was inherited by four successive unstressed generations [9].

**Figure 1:**
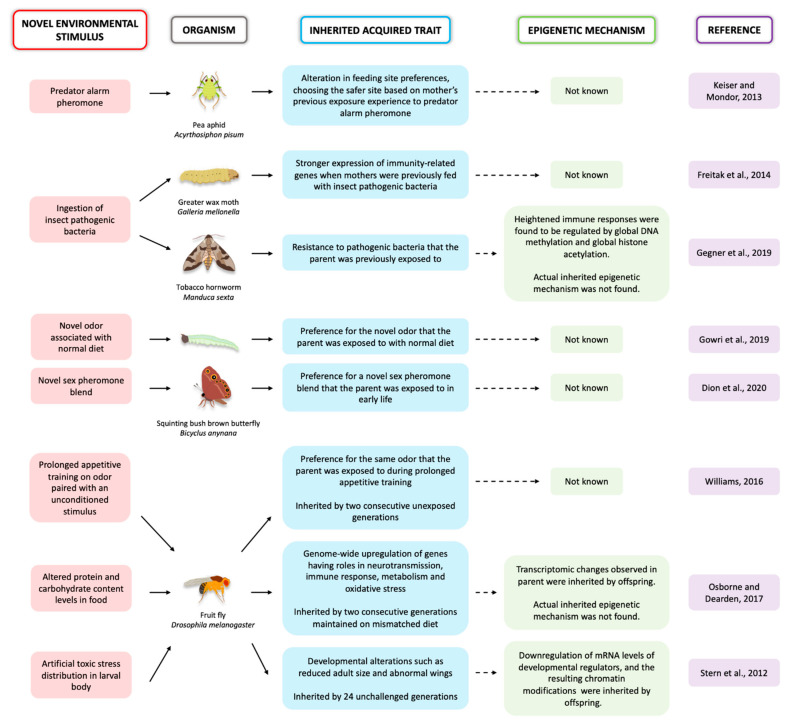
A summary of the research studies on the inheritance of acquired traits in insects discussed in this review article.

Most insect species display some form of developmental and/or behavioral plasticity, which might make them ideal model systems for the study of epigenetic inheritance if some of the mechanisms for plasticity and for epigenetic inheritance are shared. Environmental stimuli with impact on the phenotype can be vast, ranging from temperature variation, parasites, pathogens, diet, and exposure to toxic compounds such as pesticides (Figure 1). Female *Acyrthosiphon pisum* pea aphids exposed to a predator alarm pheromone produced offspring that showed altered feeding site preferences. These aphid offspring chose safer sites based on the mother’s previous experience [10]. *Galleria mellonella* moth larvae showed increased expression of immunity-related genes and increases in antimicrobial enzymes in the hemolymph when fed with insect pathogenic bacteria. The eggs laid by those females also showed stronger expression of immunity-related genes when compared to eggs laid by unexposed females [11]. In a study on *Drosophila melanogaster* flies, adults were exposed to one of two different odors and each odor was paired with an unconditioned stimulus, either sucrose (a positive stimulus) or a mild electric shock (a negative stimulus). The flies that underwent prolonged appetitive (odor coupled with sucrose) training produced first and second-generation unexposed offspring displaying a weak but a selective approach to the parent-trained odor [12]. When *Bicyclus anynana* butterfly larvae were fed with their normal diet but coated with a novel odor, they learned to prefer this novel food later in life. This learned preference was also inherited by the offspring [13]. Female *B. anynana* butterflies also learned to prefer novel sex pheromone blends when exposed to those blends early in life. This learned preference was passed down to the next generation [14].

## 2. Epigenetic Mechanisms in Animals That Play a Role in the Inheritance of Acquired Traits

The mechanisms underlying the inheritance of such environmentally induced traits may, in some cases, involve ‘epigenetics’. This term was devised by C. H. Waddington in 1942, but only gained popularity in the 1990s [15]. Waddington defined the term ‘epigenetics’ as “the branch of biology which studies the casual interactions between genes and their products, which bring the phenotype into being” [16,17]. It was an early definition that we now mostly associate with organismal development. Later, the term was redefined as “the study of changes in gene function that are heritable and that do not involve a change in DNA sequence” [18]. DNA methylation, histone modifications and small noncoding RNAs are examples of epigenetic factors that regulate gene expression in response to environmental stimuli [19,20]. Epigenetic factors are mutagenic, i.e., they are able to induce heritable genetic changes that are permanent. Thus, epigenetics is capable of stimulating and maintaining evolution in the long run [21]. However, some epigenetic marks can be temporary, leading to reversible phenotypes in the absence of the environment stimulus. Hence, it is important to identify what epigenetic mechanisms underly the transmission of acquired traits and what mechanisms allow for trait stability in the absence of the original causative environment.

Epigenetic factors can be inherited by only one generation or across multiple generations. Epigenetic inheritance is of two types, namely intergenerational epigenetic inheritance (IEI) and transgenerational epigenetic inheritance (TEI). When an environmental stress or stimulus influences changes in the epigenome of the exposed parent (F0) it can also directly affect its germ cells (F1). If, as a consequence, modified epigenetic factors are inherited by the immediate offspring (F1), it refers to IEI [22,23]. However, if the F1 offspring are unexposed to the same environmental stress or stimulus, and yet the modified epigenetic factors are found to persist in its offspring (F2) and likely beyond in the absence of further exposures, the effect is termed as TEI. In mammals, depending on the exact exposure procedure, TEI can only be claimed for effects persisting into the F3 and beyond. This is because if mothers are exposed to an environmental factor whilst pregnant their embryos (F1) carry their own germ cells (F2), which have the potential to be directly affected by the environment as well [22,23,24]. In this review, we will be discussing research studies on both inter and transgenerational inheritance of epigenetic mechanisms.

### 2.1. DNA Methylation

Several epigenetic mechanisms that underlie the inheritance of learned experiences or preferences in animals have, so far, been discovered. One of the most extensively studied mechanisms is DNA methylation [25,26]. DNA methylation is the addition of a methyl group to the pyrimidine ring of the cytosine residues (CpG dinucleotides). This forms 5-methylcytosine that directly affects chromatin remodeling and gene expression, without altering the DNA sequence [27]. The enzymes that play an important role in DNA methylation are called DNA methyl-transferases (DNMTs) [28]. DNA methylation can inhibit the transcription of genes directly or indirectly. If the gene promotors contain sites that are methylated, these can prevent DNA-binding proteins and transcription factors from interacting with the DNA at those sites. Methylated DNA can also alter chromatin structure and act as a transcriptional repressor or gene silencer by recruiting and binding to the methyl-CpG binding protein (MBD) [29,30,31].

Although DNA methylation has been studied in a number of vertebrate and invertebrate animal models, this mechanism is rare in the premier *C. elegans* and *D. melanogaster* animal model systems [31,32]. Animals that do have methylated DNA after an environmental exposure include water flees, mice, and fish. For example, exposing *Daphnia magna* (water flees) to high salinity levels, resulted in hypomethylation of genes responsible for cellular stress response maintenance. This specific pattern of methylation was found to be inherited by three consecutive generations that were not exposed to salinity stress [33]. Gestating mice, when injected with a pesticide mixture, had abnormalities in puberty, and testis and ovarian diseases in unexposed F1 and F3 offspring. Upon analysis of the F3 sperm genome, various differentially methylated DNA regions were identified [34]. A final example includes the molly fish, *Poecilia mexicana* which is capable of surviving in environments of extreme toxic levels of hydrogen sulfide. For this study, wild adult populations from both sulfidic and nonsulfidic fields were collected and grown in the lab with normal hydrogen sulfide levels for two generations. Upon comparing both populations, the researchers found stably inherited methylated DNA regions in the F2 laboratory offspring even in the absence of an extreme sulfidic environment. These regions of DNA methylation were related to various cellular activities ranging from signaling to apoptosis, and sulfur metabolism. These methylated regions showed a high degree of overlap across generations, suggesting DNA methylation as a possible epigenetic mechanism for this heritable adaptive phenotype [35].

### 2.2. Histone Modifications

Another epigenetic mechanism commonly studied in conjunction with DNA methylation is histone modification. Histone modifications comprise the addition or removal of chemical groups such as acetyl, methyl, phosphate and ubiquitin groups to the positively charged histone protein tails. These modifications change the structure of chromatin, making it open and accessible or closed and inaccessible. Only open chromatin allows transcription factors to bind regulatory regions of genes, ultimately affecting gene expression [36]. When *Artemia* brine shrimp were exposed to heat stress, this induced heat shock protein Hsp70 production, helping to develop tolerance to heat and resistance to pathogenic bacteria. These acquired adaptive defensive traits were passed down to three successive generations of unexposed offspring, favoring a significantly higher survival when compared to the offspring of the control group. The researchers found both global DNA methylation and global acetylation of histones H3 and H4 to be the epigenetic mechanisms for these heritable adaptive phenotypes [37]. In another study done on *Rattus norvegicus*, the F0 rats self-administered cocaine infusions. Reduced self-administration of cocaine was observed only in the male offspring of parents exposed to cocaine, relative to offspring sired by control parents. Analysis of the parent’s sperm using ChIP-qPCR showed an increased acetylation of histone H3 associated with the brain-derived neurotrophic factor (BDNF) promotor [38].

### 2.3. Small Noncoding RNAs

A number of recent studies have shown that a variety of small noncoding RNAs (sncRNA) are involved in epigenetic transmission across generations. Small interfering RNA (siRNA), piwi-interacting RNA (piRNA) and micro RNA (miRNA) are examples of sncRNAs [27,39]. miRNA and piRNA regulate both transcriptional and translational processes, silence genes by recruiting the machinery to specific gene promoters, silence transposons, modify proteins and also bind to mRNA targets [40,41,42]. These sncRNAs can be transferred via extracellular vesicles, called exosomes, from somatic cells to germ cells [27,39]. Studies that implicate these sncRNAs in epigenetic transmission have been performed in mice and worms.

In mice, studies have shown that physiological and behavioral changes in response to traumatic stress or odors were inherited by the offspring and were mediated by small noncoding RNAs. In the first study, researchers found that sncRNAs extracted from the sperm of mice exposed to traumatic stress and injected into wildtype fertilized oocytes led to offspring showing the same metabolic and behavioral changes that were previously observed in the stressed parents. These traumatized mice also had lower levels of piRNAs, and high levels of numerous miRNAs (related to stress response) in their sperm in comparison to the control mice [43]. In the other study, male mice were exposed to odors coupled with mild foot shocks, and the female offspring showed a heightened response to the odor. This behavioral response in the offspring was similar to that of the treated parent. Differentially expressed miRNAs that contributed to the increased sensitivity of the mice to the odor, and enhanced olfactory-related neuroanatomy, were identified in sperm from the odor-exposed parent mice relative to the control parents [44]. This study showed paternal influence in the inheritance of a learned behavior mediated by sncRNAs.

In worms, small RNAs involved in the regulation of nutrition and virus-like particles are capable of horizontally and transgenerationally transferring a learned memory. In the first study, starving *C. elegans* led to the production of small RNAs that targeted genes with nutrition-related roles. These small RNAs were inherited by three consecutive offspring generations. F3 offspring of the starved worms showed increased longevity and the inherited small RNAs orchestrated aging-related gene regulatory functions [45]. In the second study, when *C. elegans* were fed the virulent strain of *Pseudomonas aeruginosa* (PA14) and thereby exposed to a P11 small RNA, the worms learned to avoid the pathogenic bacteria using this P11 sRNA cue. The lysate from the F2 offspring of those trained worms was able to transfer the learned avoidance memory to naïve animals. This horizontally-transferred avoidance behavior was found to be transgenerationally inherited by four successive generations. Scientists showed that *Cer1* retrotransposon virus-like particles in the homogenized worm lysate were required for this horizontal transfer and transgenerational epigenetic inheritance [46].

## 3. Epigenetic Mechanisms in Insects That Play a Role in the Inheritance of Acquired Traits

Immune challenges applied to different species of moths have led to long-lasting gene expression changes on subsequent, untreated generations, but the epigenetic mechanism of inheritance is still unclear (Figure 1). When *Manduca sexta* moths were fed with the pathogenic bacteria *Serratia entomophila*, the moths showed heightened immune responses that were regulated by changes in DNA methylation as well as histone acetylation. The immediate offspring acquired resistance to these pathogens via transgenerational immune priming (TGIP). These offspring showed differential global DNA methylation and global histone acetylation that was associated with genes conferring immunity, interestingly, in a sex-specific manner [47]. The actual factors that were inherited by the offspring, and that led to these epigenetic changes, are still unknown.

In two other studies performed in fruit flies, diet was shown to alter gene expression in multiple subsequent generations but the epigenetic mechanisms that were inherited and that led to these changes are still unclear. In one study, scientists fed F0 *D. melanogaster* fruit flies with either HPLC (high-protein, low-carbohydrate) or LPHC (low-protein, high carbohydrate) diets and analysed the transcriptome of those flies and that of the three successive generations that were maintained on either HPLC diet, or the standard lab fly diet (LPHC). The F0 flies that were fed HPLC diet showed a genome-wide upregulation of genes having roles in neurotransmission, immune response, metabolism and oxidative stress, in comparison to flies that were fed LPHC diet. The transcriptomic changes observed in offspring that were maintained on a mismatched diet (LPHC) were inherited at moderate levels by F1 and F2 but not by F3 offspring, when they reverted back to F0 levels. These observed changes showed that altered nutrition can have multigenerational effects on the transcriptome [48]. The second study also used fruit flies with the goal of exploring how organisms cope with sudden, unfamiliar environmental challenges, such as toxic stress, and examined resulting transgenerational effects. Scientists created an artificial distribution of toxic stress in the larvae. They did this by expressing the *neoGFP* resistance gene under randomly chosen tissue-specific developmental promotors, and fed those larvae lethal concentrations of G418, an aminoglycoside antibiotic drug, along with the lab diet. This led to drastic changes in fly development such as reduced adult size and abnormal wings. These modifications in development were facilitated by the downregulation of mRNA levels of Polycomb genes, which are important development regulators. These changes, in turn, drove modifications in the chromatin, and this response was inherited by as many as 24 unchallenged generations [49].

## 4. Genetic Assimilation

The inheritance of acquired traits, upon exposure to novel environmental stimuli, can become fixed, and long-lasting, via natural selection. Conrad H. Waddington in 1957 coined the term ‘genetic assimilation’, which refers to the evolutionary process of transferring an environmental induction of a phenotype to a genetic induction. After that transfer, the phenotype becomes expressed even in the absence of the initial causative environmental stimulus. Genetic assimilation was first proposed in a study of bithorax-like phenocopies in *D. melanogaster*. When fly embryos were treated with ether vapor, a fraction of the embryos developed enlarged halteres in the 3rd thoracic segment, resembling a second pair of wings or a double thorax. Upon repeated selection for this “bithorax” phenotype, *D. melanogaster* with wing-like halteres were found among untreated individuals in fewer than 30 generations [50]. When this experiment was repeated in 1996, genetic variants at the *Ubx* locus were shown to be selected alongside the more extreme bithorax phenotypes, suggesting that such genetic variation contributed to the mechanism of genetic assimilation [51]. In another study, when *Caenorhabditis remanei* nematodes were exposed to a nonlethal high temperature during the early larval stage, they showed higher heat-shock resistance and reduced mortality upon exposure to a heat-shock later in life when compared to worms that were not exposed to the high temperature during their early development. When individuals showing this heat-resistance were selected over 10 generations, the worms evolved a high heat withstanding capability even in the absence of an initial heat exposure [52]. In another experiment, *C. elegans* worms were exposed to different dilutions of new odors benzaldehyde or citronellol in their food environment, over the course of five generations. Upon analyzing their chemotaxis behavior, they found that the nematode worms moved towards the same odor for the next 40 generations, even without any further imprinting. This showed that the learned behavioral response to a novel odor had been genetically assimilated in that worm population [53]. These studies show that genetic assimilation is a powerful evolutionary mechanism, but the underlying genetic and epigenetic mechanisms of this phenomenon are still largely unexplored.

## 5. Conclusions

There is substantial research on insects showing that they can modify their phenotype or their behavior in response to novel environmental stimuli such as diet, odors, pheromones, and temperature, and overcome the Weismann’s barrier by directly transmitting the modified trait from the parental soma to their offspring through the germline. However, the mechanisms underlying the inheritance of such acquired traits are still largely unexplored. This is interesting because the known trait modifications observed in insects suggest that the modified trait, especially behaviors, aid their survival. Some of these behaviors, such as altered preferences for odors or pheromones, develop in the brain. However, these altered behavioral phenotypes are eventually encoded, in some format, in the germline and reappear in their offspring. The mechanisms for this type of behavioral inheritance are not known for insects. Modifications to DNA methylation, histone acetylation, and sncRNAs are all inheritance mechanisms discovered in other animal systems. These mechanisms might all play a role in the transmission of acquired traits in insects, and thus it is important to analyze both DNA and RNA-based data in such studies for more insight into the underlying inheritance mechanisms. Finally, the ability to rear insects in large numbers, with a lower cost compared to mammals, as well as their high fertility and short generation times, will simplify future investigations of transgenerational effects and the inheritance of acquired traits in animals.

## References

[1] Yawen Z. (2014). Charles Darwin’s Theory of Pangenesis. Embryo Project Encyclopedia.

[2] Yawen Z. (2015). The Germ-Plasm: A Theory of Heredity (1893), by August Weismann. Embryo Project Encyclopedia.

[3] Tollrian R. (1990). Predator-induced helmet formation in *Daphnia cucullata* (Sars). Arch. FürHydrobiol..

[4] Tollrian R. (1995). *Chaoborus crystallinus* predation on *Daphnia pulex*: Can induced morphological changes balance effects of body size on vulnerability?. Oecologia.

[5] Waterland R.A., Jirtle R.L. (2003). Transposable elements: Targets for early nutritional effects on epigenetic gene regulation. Mol. Cell. Biol..

[6] Owen D., Matthews S.G. (2007). Prenatal glucocorticoid exposure alters hypothalamic-pituitary-adrenal function in juvenile guinea pigs. J. Neuroendocr..

[7] Crudo A., Petropoulos S., Moisiadis V.G., Iqbal M., Kostaki A., Machnes Z., Szyf M., Matthews S.G. (2012). Prenatal Synthetic Glucocorticoid Treatment Changes DNA Methylation States in Male Organ Systems: Multigenerational Effects. Endocrinology.

[8] Dias B.G., Ressler K.J. (2014). Parental olfactory experience influences behavior and neural structure in subsequent generations. Nat. Neurosci..

[9] Kishimoto S., Uno M., Okabe E., Nono M., Nishida E. (2017). Environmental stresses induce transgenerationally inheritable survival advantages via germline-to-soma communication in Caenorhabditis elegans. Nat. Commun..

[10] Keiser C.N., Mondor E.B. (2013). Transgenerational Behavioral Plasticity in a Parthenogenetic Insect in Response to Increased Predation Risk. J. Insect Behav..

[11] Freitak D., Schmidtberg H., Dickel F., Lochnit G., Vogel H., Vilcinskas A. (2014). The maternal transfer of bacteria can mediate trans-generational immune priming in insects. Virulence.

[12] Williams Z.M. (2016). Transgenerational influence of sensorimotor training on offspring behavior and its neural basis in Drosophila. Neurobiol. Learn. Mem..

[13] Gowri V., Dion E., Viswanath A., Piel F.M., Monteiro A. (2019). Transgenerational inheritance of learned preferences for novel host plant odors in *Bicyclus anynana* butterflies. Evolution.

[14] Dion E., Pui L.X., Weber K., Monteiro A. (2020). Early-exposure to new sex pheromone blends alters mate preference in female butterflies and in their offspring. Nat. Commun..

[15] Deichmann U. (2016). Epigenetics: The origins and evolution of a fashionable topic. Dev. Biol..

[16] Waddington C.H. (1968). Towards a Theoretical Biology. Nature.

[17] Urvalek A., Laursen K.B., Gudas L.J. (2014). The Roles of Retinoic Acid and Retinoic Acid Receptors in Inducing Epigenetic Changes. Subcell. Biochem..

[18] Wu C.-T. (2001). Genes, Genetics, and Epigenetics: A Correspondence. Science.

[19] Nègre N., Brown C.D., Ma L., Bristow C.A., Miller S., Wagner U., Kheradpour P., Eaton M.L., Loriaux P., Sealfon R. (2011). A cis-regulatory map of the Drosophila genome. Nature.

[20] Jirtle R.L., Skinner M.K. (2007). Environmental epigenomics and disease susceptibility. Nat. Rev. Genet..

[21] Danchin E., Pocheville A., Rey O., Pujol B., Blanchet S. (2019). Epigenetically facilitated mutational assimilation: Epigenetics as a hub within the inclusive evolutionary synthesis. Biol. Rev. Camb. Philos. Soc..

[22] Knudsen T.M., Rezwan F.I., Jiang Y., Karmaus W., Svanes C., Holloway J.W. (2018). Transgenerational and intergenerational epigenetic inheritance in allergic diseases. J. Allergy Clin. Immunol..

[23] Tuscher J.J., Day J.J. (2019). Multigenerational epigenetic inheritance: One step forward, two generations back. Neurobiol. Dis..

[24] Jawaid A., Mansuy I.M. (2019). Inter- and transgenerational inheritance of behavioral phenotypes. Curr. Opin. Behav. Sci..

[25] Eli-Byarlay H. (2016). The Function of DNA Methylation Marks in Social Insects. Front. Ecol. Evol..

[26] Wellband K., Roth D., Linnansaari T., Curry R.A., Bernatchez L. (2021). Environment-driven reprogramming of gamete DNA methylation occurs during maturation and is transmitted intergenerationally in salmon. BioRxiv.

[27] Engmann O., Mansuy I.M., Chen A. (2020). Chapter 18–Stress and its Effects Across Generations.

[28] Bogdanović O., Veenstra G.J.C. (2009). DNA methylation and methyl-CpG binding proteins: Developmental requirements and function. Chromosoma.

[29] Fan G., Hutnick L. (2005). Methyl-CpG binding proteins in the nervous system. Cell Res..

[30] Du J., Johnson L.M., Jacobsen S.E., Patel D.J. (2015). DNA methylation pathways and their crosstalk with histone methylation. Nat. Rev. Mol. Cell Biol..

[31] Bommarito P.A., Fry R.C., McCullough S.D., Dolinoy D.D. (2019). Chapter 2-1—The Role of DNA Methylation in Gene Regulation.

[32] Roberts S.B., Gavery M.R. (2012). Is There a Relationship between DNA Methylation and Phenotypic Plasticity in Invertebrates?. Front. Physiol..

[33] Jeremias G., Barbosa J., Marques S.M., De Schamphelaere K.A., Van Nieuwerburgh F., Deforce D., Gonçalves F.J., Pereira J.L., Asselman J. (2018). Transgenerational Inheritance of DNA Hypomethylation in Daphnia magna in Response to Salinity Stress. Environ. Sci. Technol..

[34] Manikkam M., Tracey R., Guerrero-Bosagna C., Skinner M.K. (2012). Pesticide and insect repellent mixture (permethrin and DEET) induces epigenetic transgenerational inheritance of disease and sperm epimutations. Reprod. Toxicol..

[35] Kelley J.L., Desvignes T., McGowan K.L., Perez M., Rodriguez L.A., Brown A.P., Culumber Z., Tobler M. (2021). microRNA expression variation as a potential molecular mechanism contributing to adaptation to hydrogen sulphide. J. Evol. Biol..

[36] Maze I., Nestler E.J. (2011). The epigenetic landscape of addiction. Ann. N. Y. Acad. Sci..

[37] Norouzitallab P., Baruah K., Vandegehuchte M., Van Stappen G., Catania F., Bussche J.V., Vanhaecke L., Sorgeloos P., Bossier P. (2014). Environmental heat stress induces epigenetic transgenerational inheritance of robustness in parthenogenetic Artemia model. FASEB J..

[38] Vassoler F., White S.L., Schmidt H.D., Sadri-Vakili G., Pierce R.C. (2012). Epigenetic inheritance of a cocaine-resistance phenotype. Nat. Neurosci..

[39] Hashemian S.M., Pourhanifeh M.H., Fadaei S., Velayati A.A., Mirzaei H., Hamblin M.R. (2020). Non-coding RNAs and Exosomes: Their Role in the Pathogenesis of Sepsis. Mol. Ther. Nucleic Acids.

[40] Aravin A.A., Lagos-Quintana M., Yalcin A., Zavolan M., Marks D., Snyder B., Gaasterland T., Meyer J., Tuschl T. (2003). The Small RNA Profile during Drosophila melanogaster Development. Dev. Cell.

[41] Ghildiyal M., Zamore P.D. (2009). Small silencing RNAs: An expanding universe. Nat. Rev. Genet..

[42] Mercer T., Mattick J. (2013). Structure and function of long noncoding RNAs in epigenetic regulation. Nat. Struct. Mol. Biol..

[43] Gapp K., Jawaid A., Sarkies P., Bohacek J., Pelczar P., Prados J., Farinelli L., Miska E., Mansuy I.M. (2014). Implication of sperm RNAs in transgenerational inheritance of the effects of early trauma in mice. Nat. Neurosci..

[44] Aoued H.S., Sannigrahi S., Hunter S.C., Doshi N., Sathi Z.S., Chan A.W.S., Walum H., Dias B.G. (2020). Proximate causes and consequences of intergenerational influences of salient sensory experience. Genes Brain Behav..

[45] Rechavi O., Houri-Ze’Evi L., Anava S., Goh S., Kerk S.Y., Hannon G.J., Hobert O. (2014). Starvation-Induced Transgenerational Inheritance of Small RNAs in C. elegans. Cell.

[46] Moore R.S., Kaletsky R., Lesnik C., Cota V., Blackman E., Parsons L.R., Gitai Z., Murphy C.T. (2021). The role of the Cer1 transposon in horizontal transfer of transgenerational memory. Cell.

[47] Gegner J., Baudach A., Mukherjee K., Halitschke R., Vogel H., Vilcinskas A. (2019). Epigenetic Mechanisms Are Involved in Sex-Specific Trans-Generational Immune Priming in the Lepidopteran Model Host Manduca sexta. Front. Physiol..

[48] Osborne A.J., Dearden P.K. (2017). A ‘phenotypic hangover’: The predictive adaptive response and multigenerational effects of altered nutrition on the transcriptome of Drosophila melanogaster. Environ. Epigenetics.

[49] Stern S., Fridmann-Sirkis Y., Braun E., Soen Y. (2012). Epigenetically Heritable Alteration of Fly Development in Response to Toxic Challenge. Cell Reports.

[50] Waddington C.H. (1956). Genetic Assimilation of the Bithorax Phenotype. Evolution.

[51] Gibson G., Hogness D.S. (1996). Effect of Polymorphism in the Drosophila Regulatory Gene Ultrabithorax on Homeotic Stability. Science.

[52] Sikkink K.L., Reynolds R.M., Ituarte C.M., Cresko W.A., Phillips P. (2014). Rapid Evolution of Phenotypic Plasticity and Shifting Thresholds of Genetic Assimilation in the Nematode Caenorhabditis remanei. G3 Genes Genome Genet..

[53] Remy J.-J. (2010). Stable inheritance of an acquired behavior in Caenorhabditis elegans. Curr. Biol..

